# Bioorthogonal Labeling Reveals Different Expression of Glycans in Mouse Hippocampal Neuron Cultures during Their Development

**DOI:** 10.3390/molecules25040795

**Published:** 2020-02-12

**Authors:** Diana Soares da Costa, João C. Sousa, Sandro Dá Mesquita, Nevena I. Petkova-Yankova, Fernanda Marques, Rui L. Reis, Nuno Sousa, Iva Pashkuleva

**Affiliations:** 1 3B’s Research Group, I3Bs – Research Institute on Biomaterials, Biodegradables and Biomimetics, University of Minho, Headquarters of the European Institute of Excellence on Tissue Engineering and Regenerative Medicine, AvePark, Parque de Ciência e Tecnologia, Zona Industrial da Gandra, Barco, 4805-017 Guimarães, Portugal; nipetkova@chem.uni-sofia.bg (N.I.P.-Y.); rgreis@i3bs.uminho.pt (R.L.R.); 2 Life and Health Sciences Research Institute (ICVS), School of Medicine, University of Minho, Campus Gualtar, 4710-057 Braga, Portugal; jcsousa@med.uminho.pt (J.C.S.); sd8tf@virginia.edu (S.D.M.); fmarques@med.uminho.pt (F.M.); njcsousa@med.uminho.pt (N.S.); 3 ICVS/3B’s—PT Government Associate Laboratory, 4710-057 Braga, Guimarães, Portugal; 4 The Discoveries Centre for Regenerative and Precision Medicine, Headquarters at University of Minho, Avepark, Barco, 4805-017 Guimarães, Portugal

**Keywords:** glycosylation, bioorthogonal chemistry, neuronal development, imaging

## Abstract

The expression of different glycans at the cell surface dictates cell interactions with their environment and other cells, being crucial for the cell fate. The development of the central nervous system is associated with tremendous changes in the cell glycome that is tightly regulated. Herein, we have employed bioorthogonal Cu-free click chemistry to image temporal distribution of different glycans in live mouse hippocampal neurons during their maturation in vitro. We show development-dependent glycan patterns with increased fucose and decreased mannose expression at the end of the maturation process. We also demonstrate that this approach is biocompatible and does not affect glycan transport although it relies on an administration of modified glycans. The applicability of this strategy to tissue sections unlocks new opportunities to study the glycan dynamics under more complex physiological conditions.

## 1. Introduction

Glycans displayed at the cell surface determine the cell interactome and fate [1,2]. In the nervous system, glycoconjugates play central roles in development, regeneration and synaptic plasticity [3,4]. They participate in the formation of a complex molecular network (both at the cell surface and in the extracellular matrix) that mediates recognition processes and triggers specific pathways. Their fundamental role in the central nervous system (CNS) is evidenced by the neuropathological and psychomotor incapacities of patients with congenital glycosylation diseases [5,6,7]. 

The structural diversity of glycans provides a myriad of possible combinations that allow fine regulation of the cell interactome: glycosylation patterns are cell-specific and in the brain, they are tightly regulated during different development stages [4,8]. Therefore, approaches that allow to monitor, control and modify glycan expression on cells are powerful tools to understand and regulate the cell fate. The roles of specific proteins in dynamic cellular processes are typically elucidated by using monoclonal antibodies and genetic fluorescent protein fusions. Analytical techniques for glycan profiling are not as straightforward as those for proteins because glycans biosynthesis is not genetically encoded and glycans structure can vary in function of the cellular environment. Therefore, imaging and quantitative analysis are extremely difficult to perform using conventional biochemical methods [9]. Bioorthogonal chemistry was introduced by Bertozzi in the early 2000s and allows biomolecule tracking in living systems without disturbing the natural biochemical processes [9,10]. In particular, metabolic glycoengineering (MGE) is a powerful method that overcomes the aforementioned limitations in the glycan analysis [11]. MGE allows the study of the cell glycorepertoire via introduction of unnatural sugars, so-called bioorthogonal reporters, into native glycans without detriment of cell physiology due to the promiscuity of the enzymes of the Roseman–Warren pathway [12]. The use of azides and alkynes as reactive moieties in bioorthogonal reporters has fostered advances in glycan imaging [13]. Both functional groups are small, biologically inert, and capable of reacting selectively with exogenous agents bearing complementary functionality at physiological pH, thus, allowing the labeling of the biomolecule with fluorophores or affinity tags [14]. Azide groups can react with the complementary phosphines via the Staudinger ligation [15,16], linear alkynes via the Cu-catalyzed azide–alkyne cycloaddition (CuAAC) [16,17], and cyclooctynes via the strain-promoted azide–alkyne cycloaddition (SAAC), also termed ‘‘Cu-free click chemistry’’ [16,17,18,19,20]. MGE has been successfully applied for in situ imaging of glycans in different cell lines and living organisms [21,22,23,24,25,26,27,28].

Despite the crucial role of glycans during neuronal development, the metabolic labeling and imaging of glycan structures in primary neurons have been reported only recently using CuAAC protocol [29]. Herein, we describe an alternative Cu-free protocol applied to mouse hippocampal neuron cultures [15,30]. The main advantage of using Cu-free click chemistry is to evade the copper-associated toxicity in biological systems [19]. We used three different reporters, namely azido-modified N-acetyl derivatives of mannosamine and glucosamine and azido-modified fucose, in combination with bioorthogonal coupling with a labeled cyclooctyne derivative to image the expression of glycans during different stages of the neuron development. We demonstrate that this protocol is a fast and reliable approach for in situ characterization of glycans in neurons.

## 2. Results and Discussion

### 2.1. Metabolic Labelling of Mouse Hippocampal Neuronal Cultures

Unlike the other biomacromolecules (polynucleotides and proteins), carbohydrates are not a genetic product. Thus, unnatural metabolic precursors can be interspersed in the carbohydrate biosynthetic pathways [31,32]. The incorporation of unnatural monosaccharides bearing reactive functional groups into cell-surface glycoconjugates provides a scenario in which the glycan can be further elaborated with an exogenously delivered imaging reagent [31]. The selectivity and rate of the reaction between the imaging agent and the incorporated carbohydrate determine the success of this strategy. Thus, the choice of the functional group/labelling reaction is crucial [20]. Among different possibilities, the copper-free azide–alkyne cycloaddition and the Staudinger ligation are better options for studies involving living cells and organisms. Herein, we have selected the copper-free azide–alkyne cycloaddition as the product stability and reaction rate of the Staudinger ligation can be compromised under in vivo conditions [33]. We prepared N-azidoacetylmannosamine (ManNAz), N-azidoacetylglucosamine (GlcNAz) and 6-azidofucose following previously described procedures (Scheme S3) [10,11,12,13,14,15,16,17,18,19,20,21,22,23,24,25,26,27,28,29,30,31,32,33,34,35,36]. The membrane penetration of unnatural metabolites is a key point of this approach and thus, the compounds were peracetylated to improve their uptake by the cells. 

**Figure 1:**
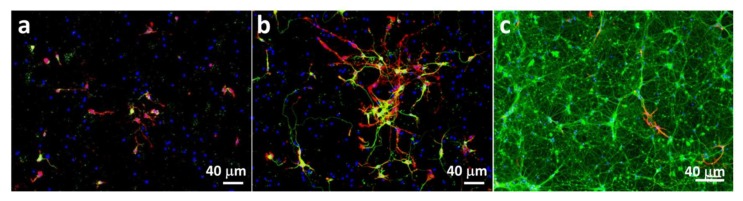
Immunofluorescence images of neuroprogenitor cells after different culture times: (**a**) 1 day, (**b**) 3 days and (**c**) 14 days. β actin is represented in red (**a**,**b**); βIII Tubulin in green (**a**–**c**) and glial fibrillary acidic protein (GFAP) in red (**c**).

The obtained unnatural metabolic precursors were feed into the culture medium of mouse hippocampal cells. To select the time points at which the supplementation would be performed, we first studied the differentiation of neuroprogenitor cells into neurons by immunofluorescence with key neuron markers (β actin, βIII Tubulin) and glial fibrillary acidic protein (GFAP) as an astrocyte marker. We observed a differentiation process up to 14 days (Figure 1). At this time point mainly neurons (Figure 1c, βIII Tubulin staining in green) and very few astrocytes are visible (Figure 1c, GFAP labeling in red) and thus, we selected it as an end point for our experiments. Because at day 3 very few cells were positive for βIII Tubulin (Figure 1b), we selected day 7 as an intermediate timepoint for metabolic labeling. Furthermore, the choice for the 7 and 14 days was made in order to avoid overcrowded cultures because: i) it would be harder to image individual cells as well as cell to cell contact; ii) more cells could split between them the sugars thus lowering the signal.

At day 7 and 14, cultures were supplemented with the unnatural metabolic precursors containing azido groups. Cells were allowed to metabolize the supplemented carbohydrates for 24 h and then the labelled cyclooctyne was introduced to initiate the click reaction. We tested different reaction conditions and found that for the studied cell cultures cyclooctyne concentrations of 50 µM and reaction time of 1 h gave the optimal output.

**Figure 2:**
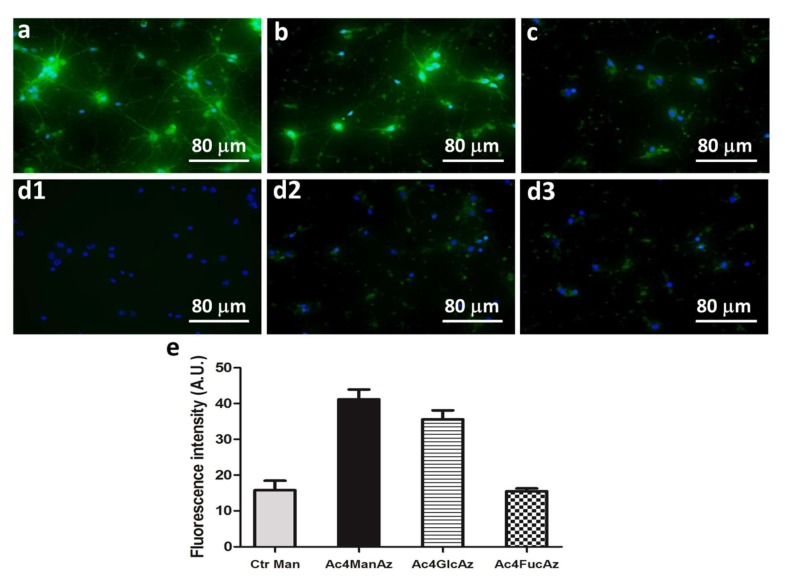
Metabolic labeling (green) of hippocampal cell cultures at day 7: cultures supplemented with peracetylated azido-functionalized (**a**) mannose (Ac_4_ManAz), (**b**) glucose (Ac_4_GlcAz) and (**c**) fucose (Ac_4_FucAz) followed by the click imaging molecule dibenzylcyclooctyne-Fluor 488/561 (DBCO-488/561, 50 µM) (**d**) Control conditions: (**d1**) cell culture without supplementation, (**d2**) culture supplemented only with the DBCO-488/561 (50 µM), (**d3**) culture supplemented with unmodified mannose and DBCO-488/561 (50 µM). Nuclei were stained with 4′,6-diamidino-2-phenylindole (DAPI) and are shown in blue. (**e**) The graphic represents fluorescence intensity measured with Fiji software (v1.50e) using representative images for the different conditions.

The supplementation of the azidocarbohydrates resulted in different fluorescence intensity among the tested carbohydrates and culture times. At day 7, highest intensity is visible for the cells supplemented with Ac_4_ManAz (Figure 2a), lower for the Ac_4_GlcAz supplemented cultures (Figure 2b) and a faint signal for Ac_4_FucAz that reveals less fucose at this stage (Figure 2c). Fluorescence is visible along the cell body but also throughout the dendrites for cells supplemented with mannose and glucose analogs (Figure 2). 

**Figure 3:**
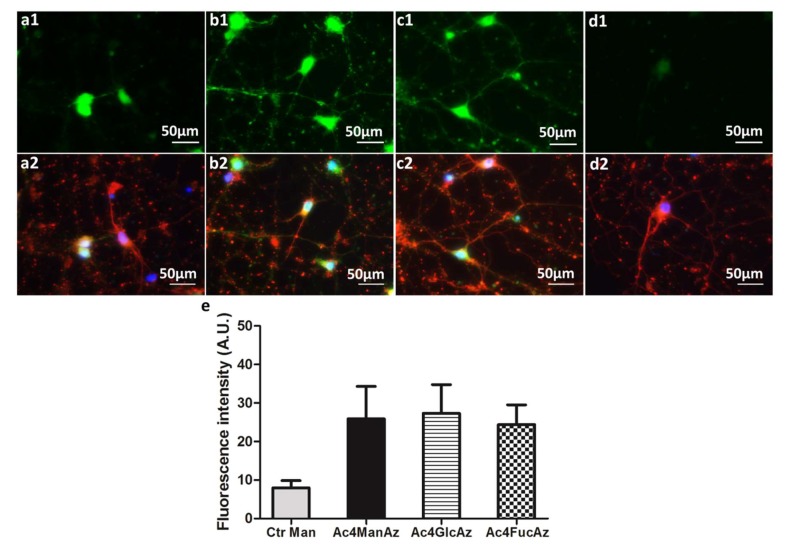
Hippocampal cells after 14 days in culture: (**a1**–**c1**) metabolic labelling of (**a1**) Ac_4_ManAz, (**b1**) Ac_4_GlcAz and (**c1**) Ac_4_FucAz followed by DBCO-488/561 (50 µM); (**a2**–**c2**) immunostaining of βIII tubulin for the same cultures (red) and nuclei with DAPI (blue); (**d1**,**2**) control samples supplemented with unmodified mannose and DBCO-488/561 (50 µM). (**e**) The graphic represents fluorescence intensity measured with Fiji software (v1.50e) using representative images for the different conditions.

At day 14, when the cell culture is mostly composed by differentiated cells (Figure 3a2–d2) the glycosylation pattern is different: incorporation of fucose increases and matches mannose and glucose derivative levels in neuronal glycoproteins (Figure 3a1–d1). These results indicate a decrease in the mannose and glucose derivative incorporation during the differentiation process and increase in the glycoprotein fucosylation. 

Sialylation (and more specifically polysialylation) and fucosylation are major post-translational modifications occurring in carbohydrate-carrying molecules, e.g., proteins, in the nervous system. These post-translational functionalizations are related with proliferation, migration and differentiation of neural progenitors [4]. Higher expression of mannose/glucose-containing glycoproteins at day 7 might indicate abundant sialylation as either mannose or glucose can be metabolized by cells to sialic acid [36]. Polysialylated Neural Cell Adhesion Molecule (NCAM) is associated with neuritogenesis and neurite outgrowth of hippocampal neurons in culture. The fact that these processes are very intensive within the first days of culture [29] can explain the results obtained with Ac4ManNAz and Ac4GlcNAz incorporation (Figure 2). While polysialylation gradually decreases [37], fucosylation increases with neuronal maturation [38]. Fucosylated glycoproteins are involved in neuronal communication. Their expression changes extensively during the course of neuronal development in mouse hippocampal tissue and during maturation of neurons in culture [38]. These previous results agree with our finding that fucose becomes more abundant with differentiation of neuroprogenitor cells (Figure 3).

### 2.2. Gene Expression Levels of Carbohydrate Transporters

**Figure 4:**
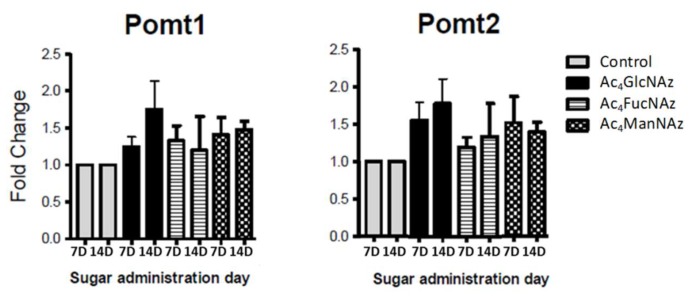
Expression of genes coding protein-*O*-mannosyltransferases (Pomt1 and Pomt2) after 7 and 14 days in culture (7D and 14D).

**Figure 5:**
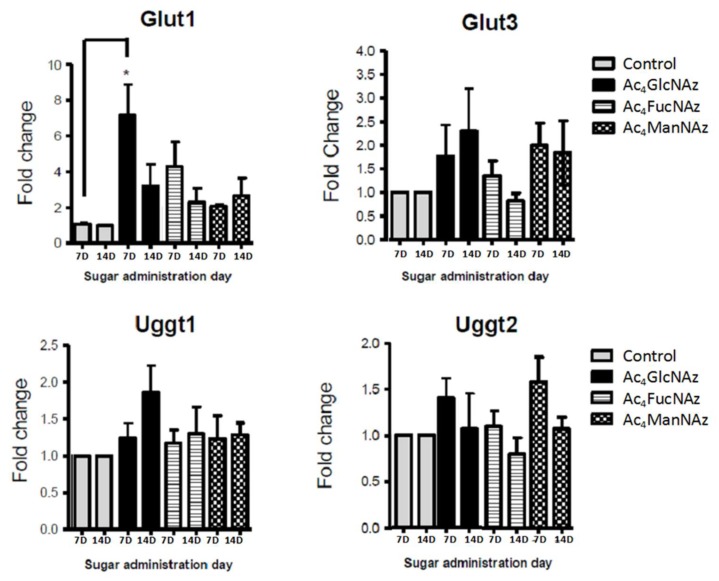
Glucose transporters and transferases gene expression after 7 and 14 days in culture (7D and 14D). * *p* < 0.05 indicates a statistically significant difference.

**Figure 6:**
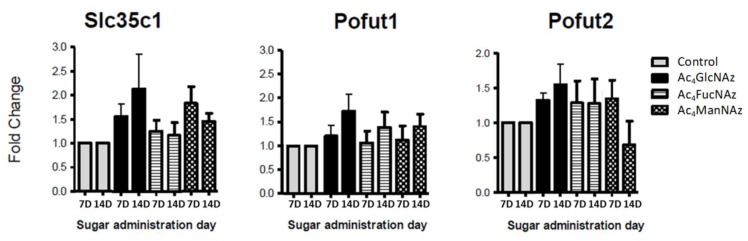
Fucose transporter and transferases gene expression after 7 and 14 days in culture (7D and 14D).

Glycosylation is incomparably crucial and, therefore, tightly controlled in neurons [4]. The formation of glycan linkage is catalyzed by highly selective glycosyltransferases (GT) with specificity both for substrate and donor nucleotide carbohydrate. The results described above showed that neuronal GT tolerate the use of unnatural azido adducts. We have used low concentration to avoid changes in the machinery used by the cell to transport and modify these molecules. To confirm that the carbohydrate transporters and transferases are not affected by these unnatural molecules, we performed RT-PCR analysis for the expression level of the respective genes (Figure 4, Figure 5 and Figure 6). 

Apart from a statistically significant (* *p* < 0.05) increase in Glut1 expression after the addition of azido modified glucose at day 7 in culture (Figure 5), no significant differences were observed for the gene expression of mannose (Figure 4) and fucose (Figure 6) transporters and transferases upon addition of azido modified carbohydrates to neuronal cell cultures. These results indicate that the used conditions (24 h period of exposition to the carbohydrate analogs at a low concentration of 50 µm) do not affect significantly the *de novo* expression of carbohydrate transporters and transferases. 

The significant change in Glut1 expression indicates that brain cells are highly responsive and sensitive to glucose fluctuations. Mammalian brain cells use glucose as a main source of energy; therefore, they depend on the tight regulation of glucose metabolism for proper physiological brain function. Disruption of the normal glucose homeostasis is the pathophysiological cause for many brain disorders [39]. In euglycemic condition, glucose concentration in the plasma is around 5–8 mM, and this corresponds to brain levels of approximately 1–2.5 mM [40]. The concentration used in this study (50 µM) is far below this range and therefore it is not expected to deleteriously affect neurons. However, the cell culture was performed in conditions of hyperglycemia (glucose concentration in Neurobasal A medium, Invitrogen is 25 mM), i.e., we used a high glucose concentration. An additional increase in the glucose concentration (by addition of azido modified glucose, Figure 6) can cause stress and be the reason for the increased expression of Glut1, as reported for adult neural stem cells [41]. 

## 3. Materials and Methods 

### 3.1. Synthesis and Characterization of bioorthogonal Reporters

*N*-azidoacetylmannosamine (ManNAz) and *N*-azidoacetylglucosamine (GlcNAz) were prepared according to a method described from Bertozzi et al. (SI, Scheme S1) [10,36]. Briefly, hydrochloride of D-aminocarbohydrate (1.0 mmol) was added to azidoacetic acid (1.37 mmol) in methanol (10 mL). After dissolution, triethylamine (0.34 mL, 2.43 mmol) was added and the reaction mixture was stirred for 5 min at room temperature (RT). The solution was cooled to 0 °C and hydroxybenzotriazole (HOBt, 0.135 g, 1.0 mmol) was added first, followed by 1-ethyl-3-(3-dimethylaminopropyl)carbodiimide (EDAC, 0.383 g, 2.0 mmol). The mixture was warmed to RT and the reaction proceeded overnight. Next, the solution was concentrated, and the residue was eluted with water over AG 50WX8 resin and AG 1-X2 resin. After concentration, the residue was further purified by silica gel chromatography, eluting with CHCl_3_–MeOH. Of note, the purification of azidoderivatives from the ammonium salt is a critical step. We have carried out the reaction of d-aminocarbohydrate with chloroacetic anhydride and used NaOH as a base (SI, Scheme S2). This approach was successfully applied for GlcNAz. In this case, a hydrochloride of D-glucosamine (1.0 mmol) was added to a suspension of NaOH (1.0 mmol) in MeOH (3 mL). The mixture was stirred at RT for 5 min and filtered. Triethylamine (0.93 mmol) and chloroacetic anhydride (4.6 mmol) were added to the filtrate. The reaction mixture was stirred for 24 h at RT. The solvent was removed and column chromatography was applied for partial purification of the compound, eluting with a gradient of CHCl_3_:MeOH (20:1 to 7:1). The resulting oil was dissolved in DMF (3 mL). NaN_3_ (3.0 mmol) was added to the solution and the reaction mixture was heated at 80 °C for 2 h. The solvent was removed and second column chromatography purification was applied, eluting with a gradient of CHCl_3_:MeOH.

The obtained azides were further peracetylated to obtain Ac_4_ManNAz and Ac_4_GlcNAz. Acetic anhydride (2.0 mL) was added to a solution of corresponding N-azidocarbohydrate in pyridine (2 mL) and the reaction mixture was stirred overnight at RT. The solution was concentrated, resuspended in CH_2_Cl_2_, and washed consecutive with 1 M HCl, saturated NaHCO_3_, and saturated NaCl. The organic phase was dried over Na_2_SO_4_, filtered, and concentrated. The crude material was purified by silica gel chromatography, eluting with hexanes–ethyl acetate (2:1, v/v). Further purification by reversed-phase HPLC (KANUER, Berlin, Germany) was also performed using column Atlantis T3 5 µm (Waters, Manchester, UK), 30 × 150 mm and eluting with a gradient of CH_3_CN and H_2_O. 

The peracetylated N-azidofucose was obtained following a different procedure (SI, Scheme S3) [35]. In the first step, we obtained 1,2:3,4-Di-*O*-isopropylidene-L-galactopyranose. Concentrated sulfuric acid (0.01 mL) was added to anhydrous zinc chloride (2.24 mmol) in dry acetone (6.5 mL). Then powdered anhydrous L-galactose (0.250 g) was added quickly, and the reaction mixture was stirred for 10 min. A suspension of Na_2_CO_3_ (0.5 g in 0.9 mL of water) was then added slowly. The suspension was filtered, and the precipitate was washed several times with acetone. Combined filtrates were evaporated under reduced pressure. The 1,2:3,4-Di-*O*-isopropylidene-l-galactopyranose was obtained from this residue after extraction with ether and purified by column chromatography (silica gel, eluent CH_2_Cl_2_:CH_3_OH 9:1). The 1,2:3,4-Di-*O*-isopropylidene-6-azido-l-galactopyranose was obtained in the next step. The 1,2:3,4-Di-*O*-isopropylidene-l-galactopyranose was dissolved in dry CH_2_Cl_2_ (2 mL). We then added dry pyridine (1 mL) and trifluoromethanesulfonic anhydride (0.372 mL). This reaction mixture was stirred for 30 min at 0 °C. After this time, the mixture was diluted with CH_2_Cl_2_ (10 mL), washed with ice-cold water (2 × 10 mL), dried over Na_2_SO_4_, filtered, and evaporated. The obtained residue was dissolved in dry DMF (1 mL) and sodium azide (0.36 g) was added. The reaction was carried at RT under stirring for 15 h. At the end of the reaction time, CH_2_Cl_2_ (10 mL) was added and the mixture was washed with water several times. After removal of the solvent we obtained a syrup-like product, which was purified by column chromatography (silica gel, eluent CH_2_Cl_2_/CH_3_OH 9:1). The purified 1,2:3,4-Di-*O*-isopropylidene-6-azido-l-galactopyranose was dissolved in CF_3_COOH/water (9:1, 5 mL), stirred for 15 min at RT and then neutralized with (CH_3_CH_2_)_3_N. After concentration, C_6_H_5_CH_3_ was added to the residue and re-evaporated. The residual syrup was acetylated with (CH_3_CO)_2_O (2 mL) in pyridine (2 mL) for 15 h at RT. The acetylated mixture was diluted with CH_2_Cl_2_ (10 mL) and washed with cold 1 N HCl, saturated NaHCO_3_, and finally with water. Solvent was evaporated and the 6-azido-1,2,3,4-tetra-*O*-acetyl-6-deoxy-l-galactopyranose was purified by HPLC chromatography. All final products were characterized by ^1^H-NMR (Bruker, Karlsruhe, Germany) and HPLC (KNAUER, Berlin, Germany). 

### 3.2. Hippocampal Neuron Isolation, Characterization and In Vitro Culture

Brains from postnatal day 1 (PND1) mice were used to obtain neurons as previously described [41]. Briefly, hippocampi were dissected, under a conventional light microscope (SZX7, Olympus, Hamburg, Germany), into smaller fragments, trypsinized for 30 min at 37 °C and mechanically dissociated through a 2 mL pipette and a Pasteur pipette. After that, the hippocampal cells were washed 5 times with Hanks’ balanced salt solution (HBSS) supplemented with 0.5% penicillin-streptomycin (Sigma-Aldrich, St. Louis, MO, USA), 10 mM HEPES solution and 1% sodium pyruvate (Invitrogen, Carlsbad, CA, USA) and re-suspended in minimum essential medium (MEM, Invitrogen) supplemented with 10% FBS, 0.5% glucose (Sigma-Aldrich), 0.5% penicillin-streptomycin, 2 mM l-glutamine and 1% MEM vitamins (Invitrogen). Hippocampal cells were plated on culture wells coated with poly-l-ornithine (Sigma-Aldrich), at a density of 50000 cells/cm^2^ and left at 37 °C in a humid atmosphere (5% CO_2_) for 5 h. After this, the medium was changed into Neurobasal A (Invitrogen) supplemented with 0.5 mM l-glutamine and 2% B27 (Invitrogen). The culture medium was changed 24 h after for Neurobasal A with 2% B27, 1% newborn calf serum (Invitrogen), 0.5 mM l-glutamine, 0.03 µM uridine (Sigma-Aldrich), 0.07 µM FDU (Sigma-Aldrich) and 1 µM kynurenic acid (Sigma-Aldrich), to prevent the proliferation of cells undergoing mitotic division and to reduce enhanced synaptic transmission. The hippocampal neurons were maintained in culture for at least 14 days, in a humid atmosphere (5% CO_2_) at 37 °C.

After 1, 3 and 14 days in culture, cells were probed for β actin, βIII Tubulin (neuron marker) and GFAP (astrocyte marker) and nuclei were counterstained with DAPI. For that, cells were fixed in 4% paraformaldehyde at RT for 20 min. Cells were incubated for 1 h at RT with the primary antibodies for GFAP (1:500, Dako, Golstrup, Denmark) and βIII-TUB (1:500, Millipore Iberica, Madrid, Spain) diluted in PBS. Cells were washed and incubated with specific Alexa 488-conjugated or Alexa 594-conjugated secondary antibodies (Invitrogen) diluted in PBS (1:500) for 1 h at RT, according to the source and isotype of the primary antibodies. Cells were washed with PBS and incubated with 4,6-diamidino-2-phenylindole (DAPI, 1:1000, Invitrogen) in PBS for 5 min at RT. Finally, cells were washed with PBS, and the glass coverslips were mounted in PermaFluor mounting medium (Thermo Fisher Scientific, Fremont, CA, USA). Fluorescence analysis and image capture were performed using a conventional (BX61; Olympus) or a confocal (FV1000; Olympus) microscope.

### 3.3. Bioorthogonal Labeling of Neuroprogenitor Cells with Peracetylated Azido Carbohydrates 

Peracetylated azido-functionalized mannose (Ac_4_ManNAz), glucose (Ac_4_GlcNAz) and fucose (Ac_4_FucNAz) were dissolved in absolute ethanol (1 mM) and a 50 µL solution was added in each well of the 24 well plate and allowed to evaporate. The azido-functionalized carbohydrates were then resuspended in culture medium (1 mL, 50 µM final concentration) and added to the cell cultures at days 7 and 14. Unmodified mannose was used as a control. After 24 h, cells were washed with PBS supplemented with 2% FBS (Gibco) and incubated with dibenzylcyclooctyne-Fluor 488/561 (50 µM, DBCO-Fluor 488/DBCO-Fluor 561, Jenabioscience, Jena, Germany) for 1 h at 37 °C. Finally, cells were washed twice with PBS supplemented with 2% FBS and fixated with 10% buffered formalin for 15 min. Nuclei were counterstained with DAPI for 15 min. Imaging of the cells with labelled glycans was performed by confocal microscopy (Olympus).

### 3.4. Gene Expression of Carbohydrate Transporters by qRT-PCR

Mouse hippocampal neuronal cultures (P1) were supplemented with azido modified carbohydrates at day 7 and 14. Cells were harvested 24 h after supplementation and mRNA was isolated for quantification of gene expression of glucose transporters (Glut1 and Glut3), UDP-glucose glycoprotein glucosyltransferases (Uggt1 and Uggt 2), fucose transporter (solute carrier family 35, member c1, Slc35c1), protein *O*-fucosyltransferases (pofut1 and pofut2) and protein-*O*-mannosyltransferases (Pomt1 and Pomt2). Total RNA was extracted from cells using the RNeasy^®^ Plus Micro Kit (Qiagen, Hamburg, Germany), following the manufacturers’ instructions. RNA quality and quantification were assessed in the NanoDrop^®^ ND-1000 (Thermoscientific, Massachusetts, USA) and 500 ng of RNA from each sample was reverse transcribed into cDNA using the iScriptTM cDNA Synthesis Kit (Bio-Rad Laboratories, Hercules, CA, USA) following the manufacturers’ instructions. Primers used to measure the expression levels of selected mRNA transcripts of *Mus musculus* by qRT-PCR were designed using the Primer3 software, on the basis of the respective GenBank sequences (Table S1). The reference gene hypoxanthine guanine phosphoribosyl transferase (Hprt) was used as internal standard for the normalization of the selected transcripts’ expression. qRT-PCR was performed on a CFX 96TM real-time system instrument (Bio-Rad), with the QuantiTect SYBR Green RT-PCR reagent kit (Qiagen) according to the manufacturer’s instructions, using equal amounts of cDNA from each sample. The cycling parameters were 1 cycle at 95 °C for 15 min, followed by 40 cycles at 94 °C for 15 s, annealing temperature (primer specific) for 30 s and 72 °C for 30 s, finishing with 1 cycle at 65 °C to 95 °C for 5 s (melting curve). Product fluorescence was detected at the end of the elongation cycle. All melting curves exhibited a single sharp peak at the expected temperature. 

### 3.5. Statistical Analysis 

Values are reported as the mean ± standard error. Each condition was tested at least in triplicates in each independent experiment and the experiments were repeated twice. Statistically significant differences between groups were determined using one-way ANOVA, followed by Tukey’s multiple comparison test. Values were considered to be statistically significant for *p* < 0.05 (*) and *p* < 0.01 (**).

## 4. Conclusions

The heterogeneity of glycans and their multiple regulatory roles, together with their importance in brain development, neuroregeneration and synaptic plasticity strongly suggests that glycans are invaluable tools to characterize neurons functions. Therefore, the development of methods to analyze the dynamics of glycan activity in neurons would be advantageous to decode their neural functions. We have proposed a neurocompatible strategy to image temporal distribution of glycoproteins during neuronal development using Cu-free click chemistry. This methodology unlocks new opportunities to study the dynamics of glycan activity in nervous system allowing decoding their effect over neuron functions. Their applicability in hippocampal tissue sections will allow in vivo tracking of glycan changes and understanding the spatial distribution of glycans within nervous tissues.

## Supplementary Materials

The following are available online. Scheme S1: Synthesis of peracetylated azidomannose (Ac_4_ManNAz); Scheme S2: Alternative synthesis of GlcNAz using chloroacetic anhydride and NaOH as a base; Scheme S3: Synthesis of peracetylated azidofucose (Ac_4_FucAz); Figure S1: ^1^H-NMR spectra of ManNAz (D2O, 300 MHz); Figure S2: ^1^H-NMR spectra of Ac_4_ManNAz (CDCl_3_, 300 MHz), mixture of anomers; Figure S3: HPLC chromatogram of purified Ac_4_ManNAz showing the two anomers; Figure S4: ^1^H-NMR spectra of GlcNAz (D2O, 300MHz); Figure S5: ^1^H-NMR spectra of Ac_4_GlcNAz (CDCl_3_, 300 MHz), mixture of anomers; Figure S6: HPLC chromatogram of purified Ac_4_GlcNAz showing the two anomers; Figure S7: ^1^H-NMR spectra of 6-azido-1,2,3,4-tetra-O-acetyl-6-deoxy-α,β-L-galactopyranose Ac_4_FucAz (CDCl_3_, 300 MHz): mixture of anomers; Table S1: Primer sequences used in qRT-PCR.
