# Supplementary material for: Bioorthogonal Labeling Reveals Different Expression of Glycans in Mouse Hippocampal Neuron Cultures during Their Development
Source: Molecules. 2020 Feb 12;25(4):795. doi: 10.3390/molecules25040795 (PMC7070308; doi:10.3390/molecules25040795)
Supplement: Supplementary file 1 [file molecules-25-00795-s001.pdf]

Supplementary Information

Bioorthogonal labeling reveals different expression of glycans in hippocampal neurons during their development

D. Soares da Costa[1,2*], J. C. Sousa[2,3], S. Dá Mesquita[2,3], N. Petkova[1,2], F. Marques[2,3], R. L. Reis[1,2,4], N. Sousa[2,3], I. Pashkuleva[1,2*]

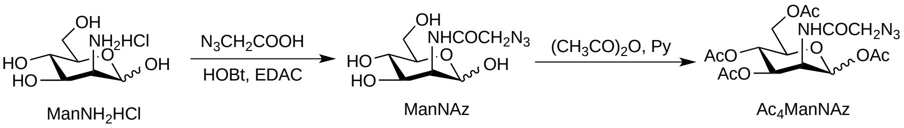

Scheme S1. Synthesis of peracetylated azidomannose ($Ac_4ManNAz$).

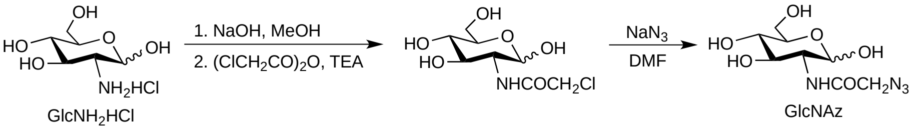

Scheme S2. Alternative synthesis of GlcNAz using chloroacetic anhydride and NaOH as a base.

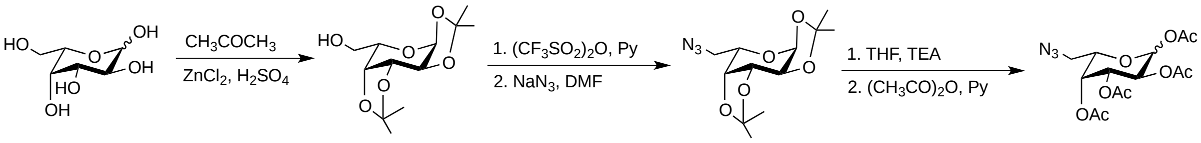

Scheme S3. Synthesis of peracetylated azidofucose (Ac_4FucAz).

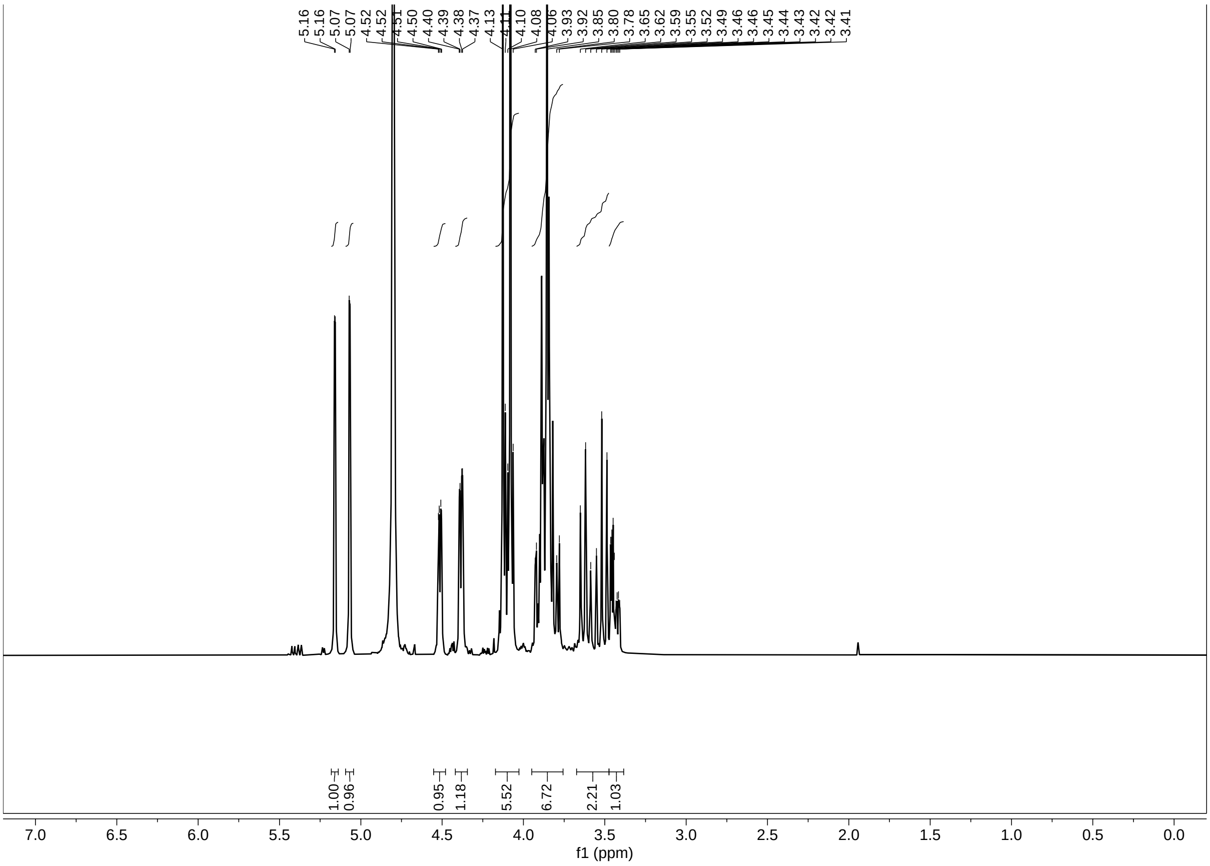

Figure S1. ^{1}H NMR spectra of ManNAz (D_2O, 300 MHz).

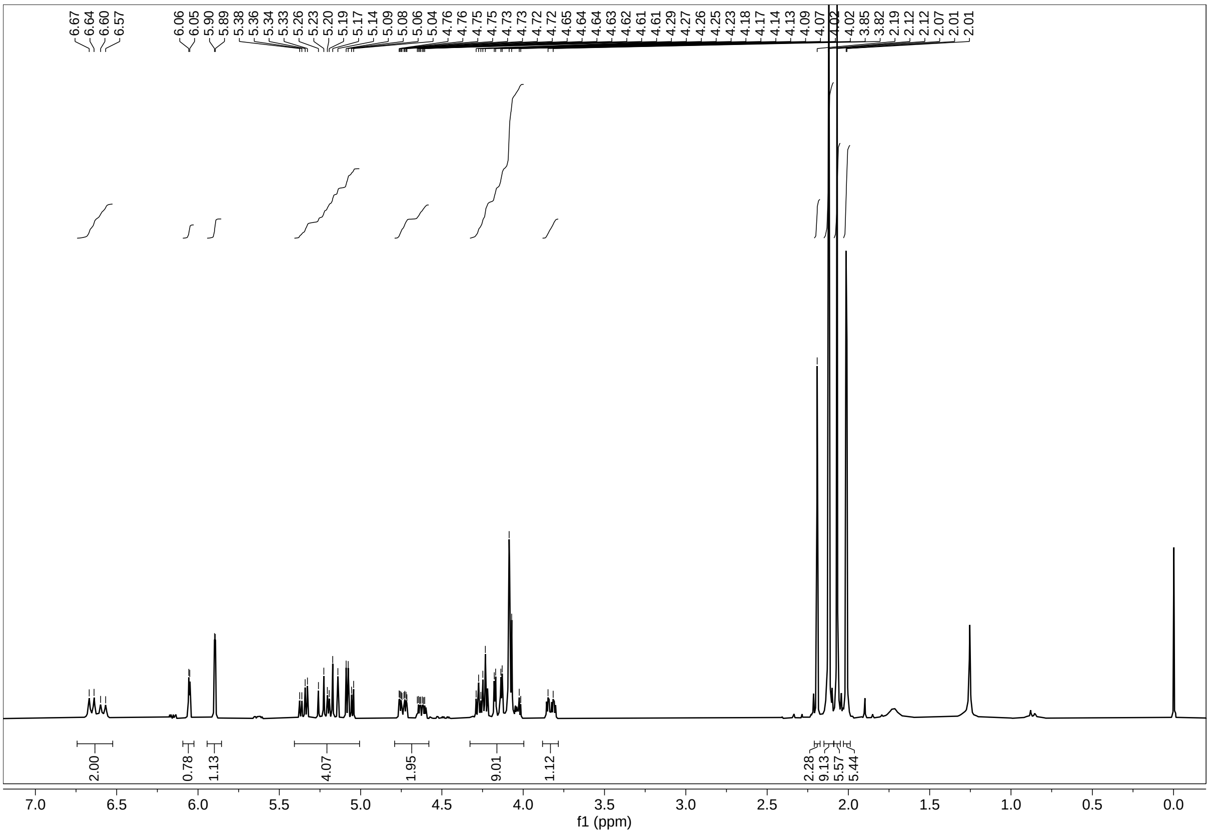

Figure S2. ^{1}H NMR spectra of Ac_4ManNAz ($CDCl_3$, 300 MHz), mixture of anomers.

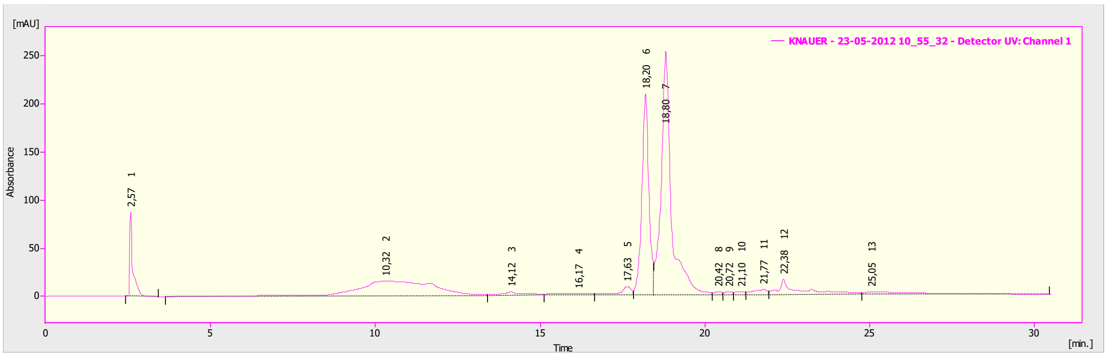

Figure S3. HPLC chromatogram of purified $Ac_4ManNAz$ showing the two anomers.

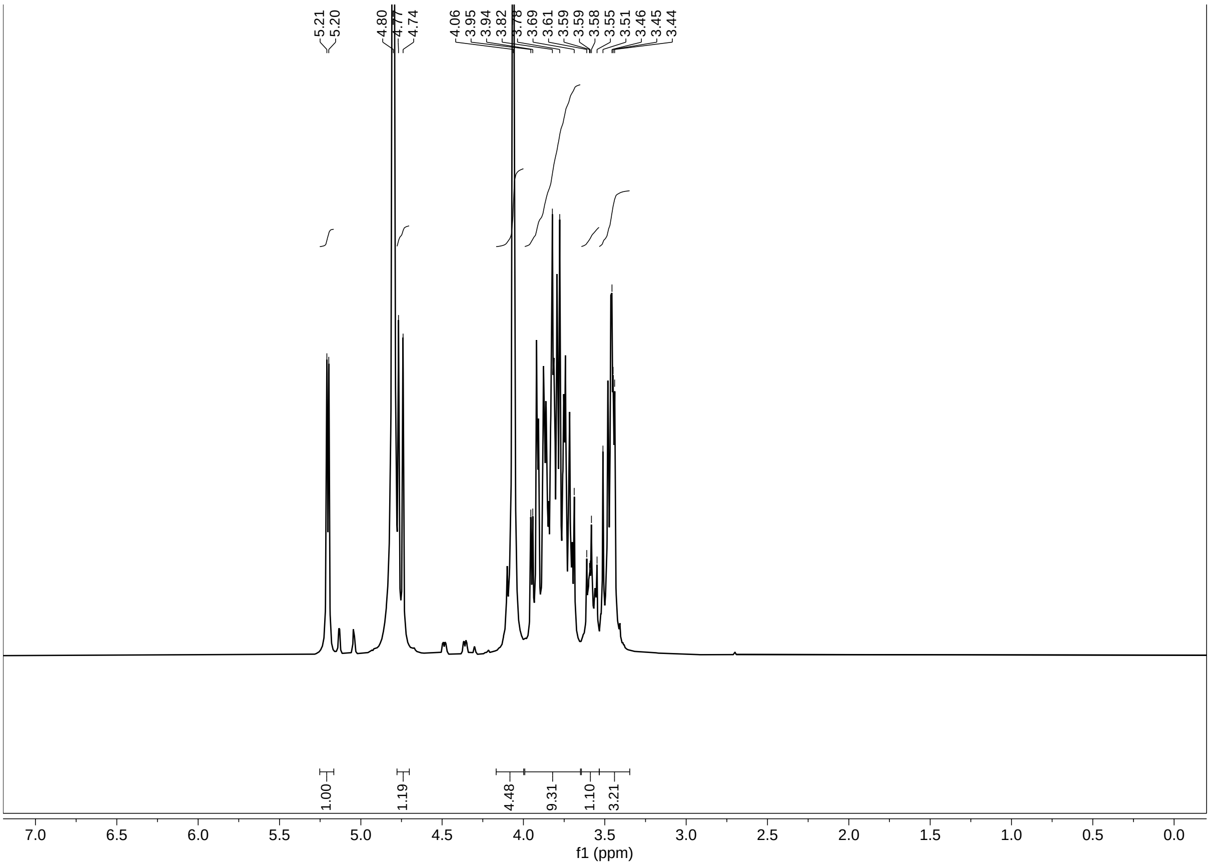

Figure S4. ^1H NMR spectra of GlcNAz (D_2O, 300MHz).

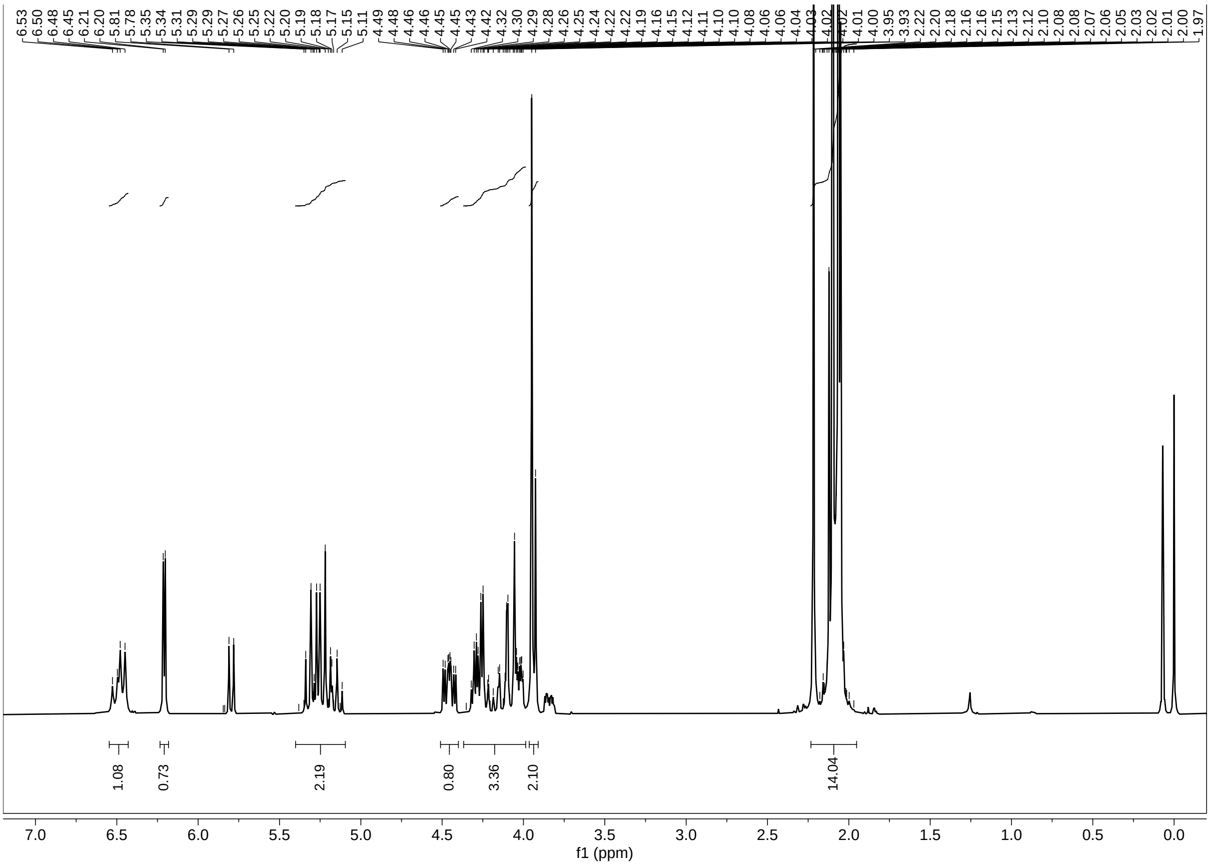

Figure S5. ^{1}H NMR spectra of $Ac_4GlcNAz$ ($CDCl_3$, 300 MHz), mixture of anomers.

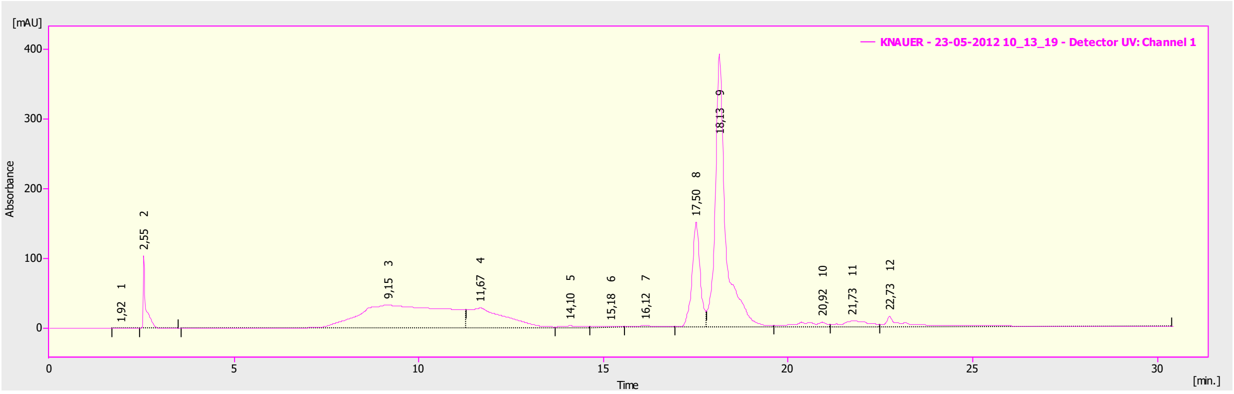

Figure S6. HPLC chromatogram of purified $Ac_4GlcNAz$ showing the two anomers.

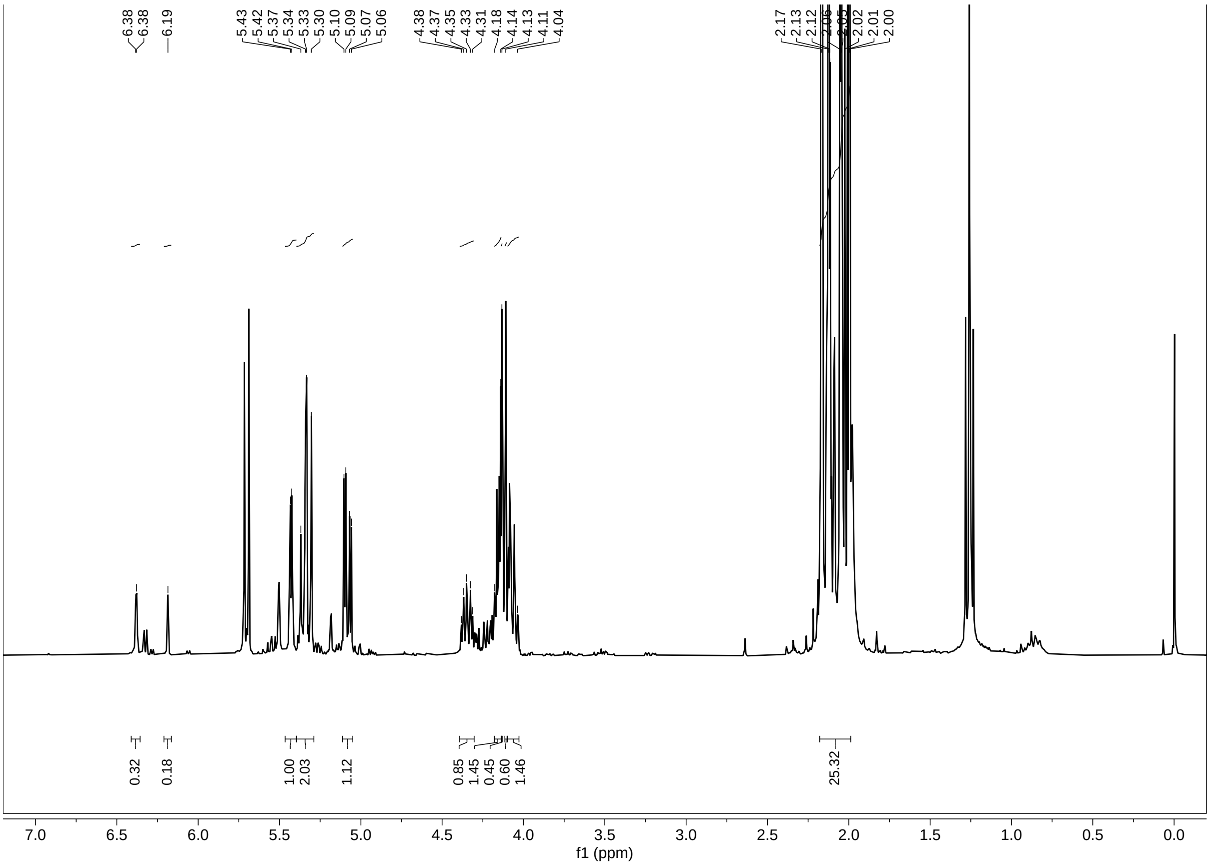

Figure S7. ^{1}H NMR spectra of 6-azido-1,2,3,4-tetra-O-acetyl-6-deoxy-α,β-L-galactopyranose Ac_4FucAz ($CDCl_3$, 300 MHz): mixture of anomers.

Table S1. Primer sequences used in qRT-PCR

Primer Name	Sequence 5'-3'
**Glut 1 F**	GTG TCG CTG TTT GTT GTA GAG C
**Glut 1 R**	CCG TGA AGA TGA TGA AGA CGT
**Glut 3 F**	TAC TCA TTG GCA TTT TCT GTG G
**Glut 3 R**	CAT CCT TCA TCT CCT GGA TCT C
**Uggt1 F**	CCT GCT ATT TCC TGG GTC AA
**Uggt1 R**	TGC TAA TCA ACT CGG CTG TG
**Uggt2 F**	CAG GGG CAA ACT CTC AGA AG
**Uggt2 R**	ACT GTT TCA ACC AGC CAT CC
**Pomt1 F**	AAT CAT GTC CAG TGC CTT CC
**Pomt1 R**	TGT TCT TGT GCG AAT GAA GC
**Pomt2 F**	TCC TGT GCC TCA TAG TGC TG
**Pomt2 R**	GCC AGG TGT TCA GGG ATA GA
**Pofut1 F**	CAG CGC CTC CTA CAA AGA AC
**Pofut1 R**	CCG TCC TCA CCA TCT CAT CT
**Pofut2 F**	CAT CAG GAA GGA GCA GGA AG

**Pofut2 R**	GTG GAA ACA GAG GTG CCA AT
**Slc35c1 F**	ATC ATT GGT GGT TTC TGG CTG
**Slc35c1 R**	AAG AAG AGC ACG CAG GCA TT
